# An Efficient System for Gene Perturbation in Embryonic Hippocampal Progenitors Using Ex Vivo Electroporation Followed by In Vitro Dissociated Cell Culture

**DOI:** 10.1177/1179069518767404

**Published:** 2018-04-19

**Authors:** Bhavana Muralidharan, Leora D’Souza, Shubha Tole

**Affiliations:** Department of Biological Sciences, Tata Institute of Fundamental Research, Mumbai, India

**Keywords:** ex vivo electroporation, dissociated cell culture, hippocampus, progenitors, gene perturbation, neurogenic factors, gliogenic factors, neuron, glia, cell fate

## Abstract

We established an efficient cell culture assay that permits combinatorial genetic perturbations in hippocampal progenitors to examine cell-autonomous mechanisms of fate specification. The procedure begins with ex vivo electroporation of isolated, intact embryonic brains, in a manner similar to in utero electroporation but with greatly improved access and targeting. The electroporated region is then dissected and transiently maintained in organotypic explant culture, followed by dissociation and plating of cells on coverslips for in vitro culture. This assay recapitulates data obtained in vivo with respect to the neuron-glia cell fate switch and can be effectively used to test intrinsic or extrinsic factors that regulate this process. The advantages of this ex vivo procedure over in utero electroporation include the fact that distinct combinations of perturbative reagents can be introduced in different embryos from a single litter, and issues related to embryonic lethality of transgenic animals can be circumvented.

## Background

Multiple factors regulate the production of neurons and glia from common progenitors in the developing cerebral cortex. Cell-intrinsic factors, including transcription factors, microRNAs (miRNAs), and chromatin-modifying factors, interact with extrinsic cues to determine the cell type produced. Initially, neurogenic pathways are active and gliogenic programs are maintained in a repressed state. This is reversed at the onset of gliogenesis, such that neurogenic pathways are repressed and gliogenic programs are activated.^1–3^

Here, we report an efficient in vitro assay for studying the regulation of the neuron-glia cell fate switch in the developing hippocampus. The procedure involves ex vivo electroporation, in which the DNA is injected into the telencephalic ventricles and the electroporation of the intact brain is performed in a petri dish. The manner in which brain is electroporated is similar to in utero electroporation. However, as the procedure is performed ex vivo, in a petri dish, the problems faced in targeting the hippocampus in utero, namely, its size which is smaller than the neocortex and its location in the caudomedial telencephalon, are not major problems because the brain can easily be oriented for optimal electroporation. Ex vivo electroporation is followed by isolation of the electroporated hippocampal primordium, dissociation of cells, and plating on coated coverslips.

This method of ex vivo electroporation permits targeting difficult structures like the hippocampus, which develops in the caudomedial wall of the developing telencephalon. This procedure takes advantage of the fact that proliferating cells inhabit the ventricular zone of the developing neural tube and therefore have the most access to DNA that is injected into the ventricle. Therefore, progenitors are best transfected using this procedure.^4^ Coupled with the use of differentiation medium, this assay is ideally suited to testing what cell types a progenitor can generate under conditions of gene overexpression or loss of function, or combinations such as overexpression of one gene combined with loss of another. The advantages of this procedure over the traditional in utero electroporation-based approach are detailed in the discussion.

### Reagents

- Ammonium chloride: Merck (catalogue number: 101145)
- B-27 supplement: Gibco (catalogue number: 17504044)
- Biotinylated goat anti–green fluorescent protein (GFP): Abcam (catalogue number: ab6658)
- DAPI: Molecular probes (catalogue number: D1306)
- Deoxyribonuclease I: Sigma-Aldrich (catalogue number: DN25)
- Ethanol: Merck (catalogue number: 107017)
- Fast green dye: Sigma-Aldrich (catalogue number: F7252)
- Fetal bovine serum: Gibco (catalogue number: 10500064)
- Fluoroshield: Sigma-Aldrich (catalogue number: F6182-20ML)
- GlutaMAX: Gibco (catalogue number: 35050-061)
- Glycine: HiMedia (catalogue number: MB013-5KG)
- Hank’s Balanced Salt Solution (HBSS): Gibco (catalogue number: 24020-117)
- Leibovitz’s L-15 medium: Gibco (catalogue number: 41300039)
- Neurobasal medium: Gibco (catalogue number: 21103-049)
- Paraformaldehyde (PFA): Sigma-Aldrich (catalogue number: P6148-5KG)
- Phosphate-buffered saline (PBS): Gibco (catalogue number: 10010023)
- Penicillin-Streptomycin 10 000 U/mL (Pen-Strep): Gibco (catalogue number: 15140-122)
- Plasmid DNA isolation kit: Macherey-Nagel (catalogue number: 740414)
- Poly-d-lysine: Sigma-Aldrich (catalogue number: P7280)
- Soybean trypsin inhibitor: Gibco (catalogue number: 17075-029)
- Streptavidin Alexa 488: Invitrogen (catalogue number: S32354)
- Triton X-100: Sigma-Aldrich (catalogue number: T8787-250ML)
- Trypan blue, 0.4%: Sigma-Aldrich (catalogue number: T8154)
- Trypsin: Gibco (catalogue number: 15090-046)

### Glassware/Plasticware

- Borosilicate glass capillaries: Sutter Instruments (catalogue number: B100-75-10)
- Cell culture 24-well plate/4-well plate: Thermo Fisher Scientific (catalogue number: 142475/176740). Well diameter ~1.5 cm, to comfortably hold the coverslip
- Cell culture 6-well plate: Thermo Fisher Scientific (catalogue number: 140675). Well diameter ~ 4 cm, to comfortably hold the Millicell filters
- Glass coverslips: Diameter 12 mm Glaswarenfabrik Karl Hecht GmbH & Co. KG (reference number: 1001-12)
- Microcentrifuge tubes: Corning (catalogue number: MCT150C)
- Millicell cell culture Insert: Merck (catalogue number: PICM03050)
- Mouth aspirator: Sigma-Aldrich (catalogue number: A5177)
- Plastic Pasteur pipettes:
  - 3 mL—Taurus Biomedical (catalogue number: P-3)
  - 2 mL—Taurus Biomedical (catalogue number: P-1)
  - 1.5 mL—Research Products International Corp. (catalogue number: 147500)
- Petri dish, 100 mm: Thermo Fisher Scientific (catalogue number: 153066)
- Petri dish, 35 mm: Laxbro (catalogue number: PD-35 TC)
- Superfrost Plus Slides: Electron Microscopy Sciences (catalogue number: 71869-10)
- Syringe filter, 0.45 μm: Millipore (catalogue number: 1211K95
- Syringe, 10 mL: BD Biosciences (catalogue number: 300294)
- Whatman filter paper: Sigma-Aldrich (catalogue number: WHA10010155)

### Equipment

- Centrifuge: Eppendorf (catalogue number: 5702000329)
- CO_2_ incubator: Heraeus HERACell 150
- Dissection tools/forceps:
  - Dumont #5 forceps: Roboz (catalogue number: RS-4978)
  - Dumont #55 forceps: Roboz (catalogue number: RS-4984)
  - Micro-dissecting Spring scissors: Roboz (catalogue number: RS-5620)
- Dual-stage Glass micropipette puller: Narishige (catalogue number: PC-10)
- Electroporator: BTX Harvard Apparatus (Model ECM 830)
- Hemocytometer: Rohem India (catalogue number: BS748)
- Horizontal flow hood: Kirloskar Envair Electrodyne (catalogue number: KCH-B)
- Micropipettes:
  - 100 to 1000 μL micropipette: Nichiryo Nichipet (catalogue number: 00-NPX2-1000)
  -  20 to 200 μL micropipette: Nichiryo Nichipet (catalogue number: 00-NPX2-200)
- Paddle electrode, 3 mm: BTX Harvard Apparatus (catalogue number: 450487)
- Stereo microscope: Olympus (catalogue number: SZ61)

### Preparation of stock and working solutions

The following reagents need to be filter-sterilized after preparation:

- Complete neurobasal media

**Table:** 

B-27 supplement	1 mL
GlutaMAX	0.5 mL
Penicillin-Streptomycin	0.5 mL
Neurobasal medium	Make up to 50 mL

- Quenching solution

**Table:** 

Glycine (2 M)	375 μL
Ammonium chloride	100 μL
PBS	Make up to 10 mL

- Poly-d-lysine solution

**Table:** 

Poly-d-lysine	5 mg
PBS	50 mL

- Trypsin inhibitor

**Table:** 

Soybean trypsin inhibitor (10 mg/mL)	140 μL
DNase I (1 mg/mL)	100 μL
HBSS	Make up to 10 mL

The following solutions do not need to be filter-sterilized:

- Blocking buffer: store at 4°C for up to 4 days.

**Table:** 

Fetal bovine serum	5 mL
Triton X-100	50 μL
PBS	Make up to 50 mL

- DAPI stock: store as aliquots of 10 to 20 μL at −20°C

**Table:** 

DAPI	10 mg
Distilled water	Make up to 1 mL

- DAPI working solution

**Table:** 

DAPI stock (10 mg/mL)	10 μL
PBS	Make up to 10 mL

- 4% PFA: can be aliquoted and frozen for extended storage, or stored at 2 to 8°C for 1 month. PFA should be prepared in a fume hood with appropriate personal protection equipment.

For 1 L of 4% PFA, place a glass beaker with 800 mL of 1× PBS on a magnetic stirrer placed in a fume hood. Maintain the temperature at 60°C. Add 40 g of PFA to the heated PBS solution and stir continuously. Add 1 N NaOH drop by drop till the powder dissolves. Cool the solution and make up the volume to 1 L with PBS. Filter using Whatman filter paper to remove particulate matter. Check the pH and adjust with small amounts of dilute HCl if it is higher than 7.4.

## Setup Prior to Starting the Procedure: Time Required Is Approximately 2 hours.

**Figure 1.**
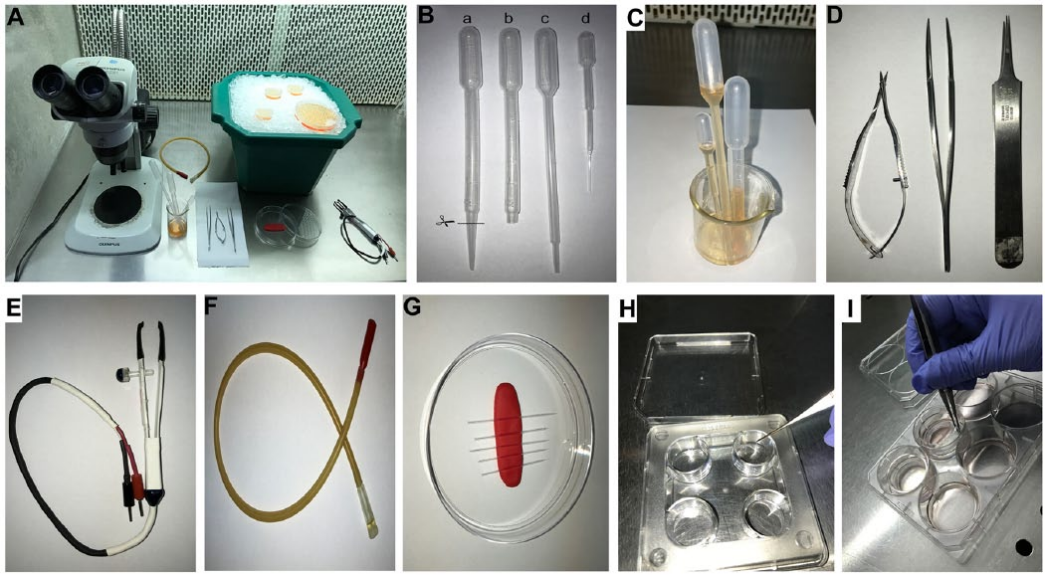
Setting up the tissue culture hood for the procedure. (A) Entire setup of materials required for the protocol, inside the laminar flow hood. (B) Pasteur pipettes “a to d” as detailed in the experimental procedure. Cut Pasteur pipette “a” at the marked position to generate Pasteur pipette “b” for transferring the embryonic brains and hemispheres. (C) Beaker with sterilized pipettes in a standing position with sterile L-15 medium, (D) forceps and micro-dissecting spring scissors, (E) 3 mm paddle electrode, (F) mouth aspirator, (G) pulled glass capillaries anchored in a petri dish, (H) placing coverslips in a 4-well plate, and (I) placing a Millicell insert in a 6-well plate.

Place a dissection microscope inside a horizontal flow tissue culture hood as in Figure 1A. Wipe both the surface of the hood and stage of the microscope with 70% alcohol. Also, set up the following items in the hood (Figure 1A):

1. Prewarm 0.25% trypsin and plating medium to 37°C and prechill PBS and L-15 medium.
2. Add ice-cold L-15 medium to 35 mm petri dishes and 100 mm petri dishes and place on the surface of a packed ice bucket (Figure 1A).
3. Prepare plastic Pasteur pipettes by cutting off a 3-mL pipette (pipette “a” in Figure 1B) to make pipette “b” so that the aperture is suited to transferring the embryonic brains and hemispheres. Also, prepare a 2-mL pipette “c” and 1-mL pipette “d” as follows: Wash all pipettes with 70% alcohol first to sterilize them and then rinse with L-15 medium to remove last traces of 70% alcohol (Figure 1B and C). Place the pipettes in a 100-mL beaker with some sterile L-15 medium (Figure 1C). The purpose of the medium is to ensure the beaker or pipettes do not topple over.
4. Sterilize forceps and micro-dissecting spring scissors with 70% alcohol and allow to dry (Figure 1D).
5. Also sterilize the paddle electrodes (Figure 1E) and mouth aspirator (Figure 1F) with 70% alcohol and allow to dry.
6. Using the capillary puller, prepare 6 pulled glass capillaries using a 1-step pull at 62.5°C. Anchor them on a sticky tape or clay in a petri dish (Figure 1G).
7. Sterilize coverslips by dipping in 100% ethanol and setting aflame using a spirit lamp as shown in Video 1: Sterilizing coverslips. Place the coverslips in a 24-well plate depending on the numbers required (Figure 1H).
8. Gently layer 250 μL of poly-d-lysine solution on top of the coverslips. Incubate at 37°C for 2 hours. Alternatively, this step can be performed the previous day and the coverslips can be incubated with poly-d-lysine at 4°C overnight. Wash the coverslips 3 times with 1 mL sterile PBS, 5 minutes per wash, followed by an additional 10-minute wash. Add 400 μL of prewarmed medium to the coverslips and place the 4-well plate in the CO_2_ incubator.
9. Using a micropipette, add 1.2 mL of complete Neurobasal medium to each well of a 6-well plate. Gently place a Millicell insert using a Dumont #5 forceps avoiding air bubbles in each well (Figure 1I). Each Millicell insert can hold approximately 6 to 8 hippocampal explants. Prepare only the required number of wells + inserts.

## Experimental Procedure

### Isolation of intact brain from mouse embryo

1. Using the procedures approved at your institution, sacrifice the pregnant dam at the desired stage, remove the embryos from the uterus, and place them in a 100-mm petri dish with prechilled sterile L-15 medium.
2. Decapitate the embryos and place the heads in a 100-mm petri dish containing chilled L-15 medium and transport on an ice bucket to the culture hood. Subsequent steps are to be performed aseptically under a dissection microscope placed in the hood.
3. Transfer one embryo head to a 35-mm petri dish containing ice-cold L-15 and gently dissect out the brain using Dumont forceps #55 and #5. The remaining embryo heads should be stored in ice-cold L-15 until step 16 is complete for the first brain.

### Electroporation and hippocampal explant preparation

**Figure 2.**
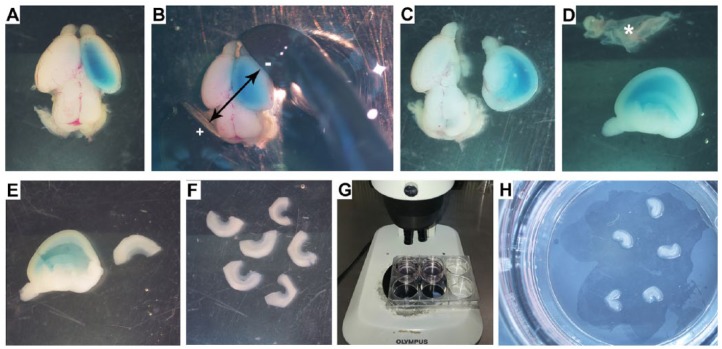
Electroporation and hippocampal explant preparation: (A) injected brain; (B) placement of the electrode to target the hippocampus; (C) dissected electroporated hemisphere; (D) removing the meninges; (E) dissecting the hippocampus from the electroporated hemisphere; (F) hippocampal explants in a petri dish; (G) 6-well plate with Millcell inserts, under the dissection microscope, for placing the explants; and (H) hippocampal explants on a Millcell insert.

4. Place the embryo dorsal side up (as shown in Video 2: Injecting DNA) and inject 3 to 4 μL of the plasmid DNA (2 μg/μL mixed with Fast green) into right lateral ventricle using a fine-glass micro-capillary. An injected brain is shown in Figure 2A.
5. Hold the 3 mm paddle electrodes at the angle/orientation and polarity as shown in Figure 2B (arrows) in order to target the hippocampus. (Please also refer to the “Potential problems and resolution” section). The user can electroporate either the right or the left hemisphere by changing the polarity and position of the electrode, but it is advisable to stick to one side to avoid confusion during the dissection of the explant.)
6. Proceed with electroporation as in Video 3: Ex vivo electroporation.

**Table:** Electroporator settings.

Voltage	45 V
Pulse length	50 ms
Number of pulses	5
Interval between pulses	1 s

7. After electroporation, transfer the brain to a fresh petri dish containing ice-cold L-15, placed on the surface of the ice bucket. Additional electroporated brains can be collected in this petri dish before proceeding to the next stage.
8. Dissect apart the electroporated hemisphere (Figure 2C). Proceed to remove the meninges using fine forceps taking care not to damage the electroporated tissue (Figure 2D; asterisk shows meninges isolated from the hemisphere).
9. Place the electroporated hemisphere medial side up and dissect out the hippocampus from the brain using micro-dissecting spring scissors (Figure 2E). Collect several medial explants in a 35-mm petri dish containing L-15 (Figure 2F).
10. Remove the 6-well plate with Millicell inserts from the incubator and place on the stage of the dissection microscope (Figure 2G).
11. Using a Pasteur pipette “c” take up the hippocampal explants together with some L-15 medium and deposit on the Millicell insert. Using Pasteur pipette (“d”) carefully remove excess media (Figure 2H). Be careful not to suck the explant into the pipette. This will cause it to lose its architecture and hence diminish cell viability.

### Dissociated cell culture

**Figure 3.**
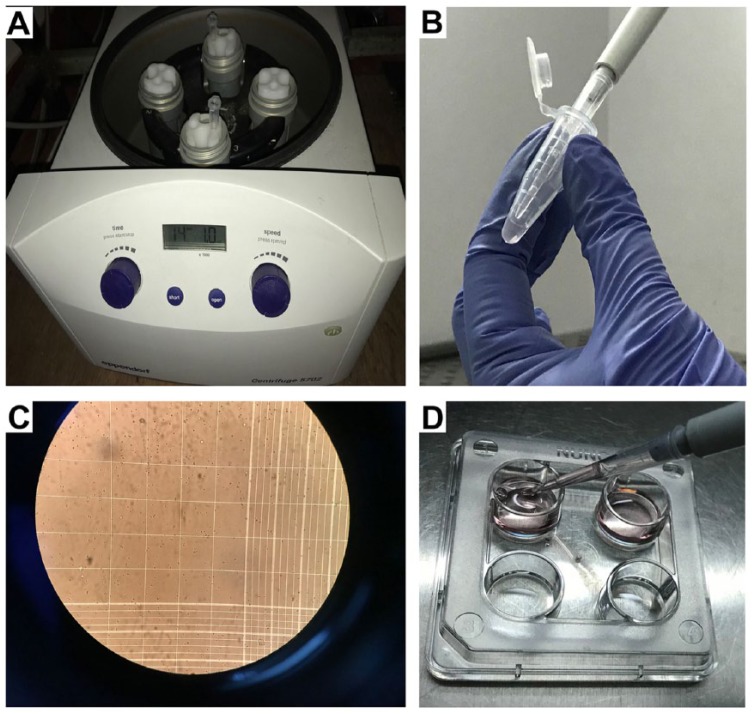
Counting and plating dissociated cells: (A) centrifugation of the explant before trituration, (B) trituration of the explant to yield single-cell suspension, (C) counting of the cells in a hemocytometer, and (D) plating the dissociated cell on precoated coverslips.

**Figure 4.**
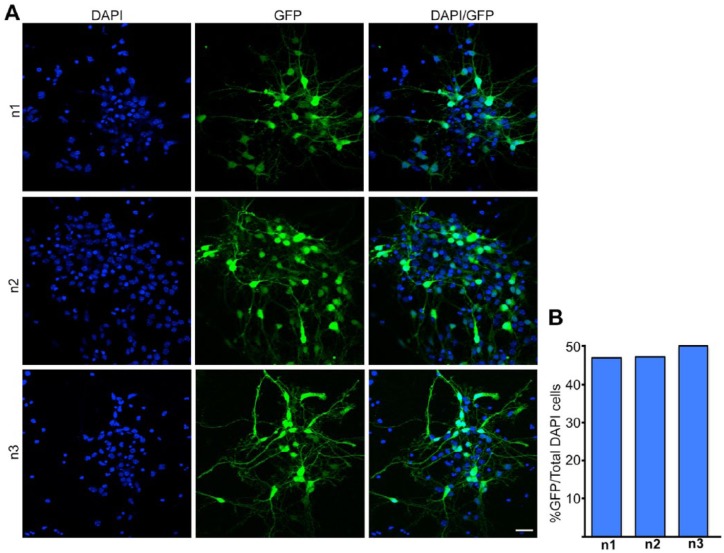
Electroporation efficiency: (A) dissociated cell cultures from 3 different E15 embryonic hippocampi electroporated ex vivo with a construct encoding *GFP* and stained with DAPI and anti-GFP antibody. (B) Quantification of the electroporation efficiency gives an average of 48% (number of GFP-expressing cells divided by the total number of DAPI-positive cells expressed as a percentage). Scale bar, 25 μm.

12. Maintain the explant on the Millicell insert in the 6-well plate for 2 hours at 37°C. This is essential for it to “recover” from the electroporation. Skipping this step drastically decreases the number of viable electroporated cells.
13. After 2 hours, add a drop of medium on the explant using a Pasteur pipette (“c”) and gently suck up the explant and transfer it to a microcentrifuge tube.
14. Remove excess medium and then add 0.4 mL of 0.25% trypsin.
15. Incubate at 37°C between 45 and 90 seconds. This step needs some standardization depending on the embryonic stage at which the tissue is isolated. The first time this assay is performed, it is ideal to test similar explants for 3 different durations of trypsin treatment and choose one that optimizes the number of dissociated single cells but does not compromise cell viability. (Please also refer to the “Potential problems and resolution” section.)
16. Add an equal volume of trypsin inhibitor solution to stop the reaction and incubate at 37°C for 2 minutes.
17. Spin down the explant at 100*g* for 5 minutes and remove the supernatant (Figure 3A).
18. Add 100 μL of Neurobasal medium and triturate each explant individually using a 200-μL pipette. Typically, one needs to triturate each sample 10 times to obtain a single-cell suspension without any visible clumps. This step needs to be standardized by each experimenter so that dissociated single cells are obtained but cell viability is not compromised (Figure 3B and Video 4: Trituration). (Please also refer to the “Potential problems and resolution” section.)
19. Add 400 μL of Neurobasal medium to the dissociated cells. Take 10 μL of the suspension and dilute 1:1 v/v with trypan blue. Count the number of cells in a hemocytometer (Figure 3C) and plate 1 × 10^5^ cells per well of the 4-well or 24-well plate containing precoated coverslips (Figure 3D).
20. If the plasmid has reporter in it, view the cells in a fluorescence microscope the next day to ascertain electroporation efficiency. In our hands, up to 48% of the cells can be electroporated (Figure 4). Change the medium in the wells every alternate day. Cells can be maintained at 37°C in a CO_2_ incubator for as many days as required for the question being tested. For the neuron-glia cell fate assay in Muralidharan et al,^5^ the cells were maintained 5 days in vitro.

### Fixing and immunostaining of dissociated cultures

All steps should be performed at room temperature on a gentle rocker, unless otherwise specified. All washes should be performed with Pasteur pipette “c” using gentle aspiration and ejection so as not to dislodge the cells:

21. Remove the culture medium and wash the cells growing on the coverslips with cold PBS (0.5 mL per wash) for 3 ×5 minutes.
22. Fix the cells in 4% (wt/vol) PFA (0.5 mL) and incubate for 20 minutes.
23. Add 0.5 mL of quenching solution and incubate for 10 minutes.
24. Wash the cells with 0.5 mL PBS for 2 × 5 minutes.
25. Incubate the cells in 0.5 mL blocking buffer for 30 minutes at 37°C.
26. Incubate the cells with 200 μL of primary antibody diluted in blocking buffer overnight at 4°C. Biotinylated goat anti-GFP was used at 1:400 dilution.
27. Wash the cells with the blocking buffer for 4 × 5 minutes.
28. Incubate the cells with the secondary antibody in blocking buffer at the mentioned dilutions at 37°C for 2 hours. For fluorescent secondary antibodies, this step should take place in the dark. Streptavidin Alexa 488 was used at 1:800 dilution.
29. Wash the cells 4 times with blocking buffer for 5 minutes each, followed by 2 washes with PBS containing 0.1% Triton-X 100.
30. Stain the cells by incubating with 0.5 mL DAPI solution for 10 minutes, followed by 2 washes with PBS 3 minutes each.
31. Gently pick up each coverslip from the well and mount it in Fluoroshield mounting medium (Video 5: Mounting coverslips).

**Table:** Timing.

Steps	Time required
Culture setup	2 h
Harvesting embryo heads	30 min
Isolating brains, electroporation, explant preparation	1 h 30 min for 6 brains + 2-h incubation
Dissociation and plating cells	45 min for 6 explants

### Plasmid design

The type of plasmid vector is important and we have summarized below some criteria that are important for successful electroporation:

1. We used plasmids in which the gene of interest (GOI) is driven under the CAG promoter, which consists of the cytomegalovirus (CMV) enhancer fused to the chicken β-actin and globin gene promoter elements. We find that CMV promoter–driven genes sometimes do not express well in expressed hippocampal cells.
2. The plasmids used should be prepared using an endotoxin-free kit to ensure good survival of cells after electroporation.
3. The plasmids should be diluted in nuclease-free water.
4. For plasmids without reporters, a separate reporter-expressing plasmid should be mixed in a 1:1 ratio to a final concentration of 2 μg/μL for electroporation.
5. The plasmid concentration mentioned is what we have used in our experiments. The users can standardize a different concentration if required.

**Table:** Potential problems and resolution.

Step	Problem	Possible reason	Solution
Electroporation (steps 4-7)	Low electroporation efficiency	(a) Improper placement of electrode leading to mis-targeting (b) Inadequate contact between electrodes and brain (c) Electrodes are worn out	(a and b) Place the electrodes as shown in the figure to target the hippocampus correctly and ensure that the brain is in contact with the electrodes during electroporation (c) Check the metal plating on the electrode surface; if corroded, replace the electrode
Dissociation (steps 12-19)	Low survival of cells	(a) The brain warmed up during and after electroporation (b) Trypsin treatment was too strong (c) Excessive trituration during dissociation of cells	(a) The brains must be kept in ice-cold L-15 wherever specified in the procedure. (b) Standardize trypsin treatment time carefully (c) Triturate gently not more than 10 times to obtain dissociated cells. If cells are not dissociated adequately, then increase trypsin treatment time

## Anticipated Results

Depending on the research question, the immunostained coverslips can be imaged using an epi-fluorescence microscope or a confocal microscope. As the cells grow in a flattened manner, there is not much information lost in the *z*-axis if the purpose is to score for a marker expressed all over the cell. However, if one wishes to adapt this protocol to study sub-cellular features such as dendritic morphogenesis or synaptogenesis,^6,7^ these are best assessed in confocal images that provide the resolution needed for detailed sub-cellular or structural analysis.

## Discussion

Current methods of genetic perturbation to examine the neuron-glia cell fate switch in vivo involve performing in utero electroporation at embryonic ages and analysis of the brains postnatally.^8,9^ However, there are several reasons why this procedure is not optimal, and there is a need for an improved, efficient procedure that utilizes fewer animals.

First, in utero electroporation procedure requires a certain level of expertise to achieve good results in terms of correct targeting and survival of pups. This requires training, and mis-targeting or prepartum/postpartum lethality of pups results in failed experiments in inexpert hands. In contrast, ex vivo electroporation requires no specialized experience beyond the ability to dissect embryonic brains. A researcher with basic tissue culture and embryonic brain dissection experience can achieve success the first time and reproducible results subsequently. Ex vivo electroporation also circumvents the constraints posed by genetically modified strains that are sensitive to anesthesia or do not respond well to surgery or have high resorption rates.

Second, in utero surgeries require stringent ethical approvals as the procedure is performed on live animals and brings with it the attendant issues of anesthesia, animal pain management, survival, and postoperative care. Ex vivo electroporation requires only standard animal sacrifice protocol clearance as the entire procedure is performed in a tissue culture dish using harvested embryonic brains. This offers an advantage for researchers who join laboratories in countries with long clearance procedures for live animal surgery.

Third, our procedure permits multiple biological replicates for several conditions in a single litter of embryos. For example, a litter consisting of 10 embryos will give one “n” for up to 10 different constructs/conditions, and 3 litters can yield n = 3 for these conditions. This is particularly useful when combinations of different constructs need to be tested. In Muralidharan et al,^5^ we used embryos from an *Lhx2lox/lox* background and electroporated either a construct encoding GFP (control) or Cre-GFP, which produced a loss-of-function phenotype. Then, in combination with Cre-GFP, we electroporated constructs that we wished to test for their ability to rescue this phenotype (Figure 3).^5^ In this manner, complex hypotheses can be tested using different combinations of constructs, with minimal usage of animals.

Fourth, for mice carrying multiple transgenes in crosses in which the proportion of embryos of the desired genotype is low, ex vivo electroporation offers the possibility of assessing embryos by phenotype where possible or building in live reporters into the genetic background, and thereby identifying the embryo with the correct genotype for the experiment. In contrast, in utero electroporation is usually performed without foreknowledge of the embryonic genotype, which is ascertained post facto after the electroporation is performed and the brains are harvested. Because rarely is it possible to electroporate 100% of the embryos in a litter, it is possible that the ones chosen for electroporation may not be those of the desired genotype.

Finally, the dissociated cell culture permits easy manipulation of the extracellular environment. Substrates can be altered, soluble factors can be added, and electroporated cells can be tested in a number of conditions and combinations that are not possible in utero.

In summary, there are several advantages to a procedure that relies on ex vivo instead of in utero electroporation.

After the step of ex vivo electroporation, it is possible to prepare slice cultures of the hippocampus, instead of dissociating the cells.^10–12^ While slice cultures have the advantage of maintaining an organotypic environment, preparing slices of E15 tissue is a challenging procedure requiring training and delicate handling skills. Examining the slices after culture is also not simple, as antibody penetration is an issue. In some cases, slice cultures need to be further sectioned on a cryostat to produce thin sections that can be examined for marker expression.^13^ In contrast, dissociation of the cells permits a clear assessment of marker expression because there are no penetration problems for the immunostaining reagents. Furthermore, epifluoresence microscopy is suitable for imaging dissociated cells, and confocal microscopy is not required. Finally, cell morphology parameters such as dendritic arborization can be scored more easily in dissociated cells.

Other standard in vitro methods involve transfection of dissociated cells using lipofectamine or transduction with viruses to deliver the gene of interest.^14,15^ In our hands, lipofectamine did not yield as many transfected cells per coverslip as we obtained with ex vivo electroporation followed by dissociated cell culture (48%) This is also supported by the literature.^6,7,14^ In the case of viral vectors, it is burdensome to construct and produce viral particles for each gene of interest. Furthermore, viral vectors require biosafety level 2 facilities, although they may be replication incompetent.

With minor modifications, this assay can be used to study additional features of neural development in vitro. For example, the use of culture medium that supports proliferation would permit the study of progenitor division, amplification, and survival.^16^ If the coverslips are maintained for longer durations, the study of neurite outgrowth and synaptogenesis is also possible.^6,7^

In Muralidharan et al,^5^ we chose E15 as the stage of assay as this is the age of peak hippocampal neurogenesis, well suited to examine whether candidate Lhx2 targets can rescue the gliogenesis arising due to loss of Lhx2. Performing this assay at earlier stages such as E12 would be suitable if the experimenter wished to examine deep layer neuronal (layer 5/6) fate specification in the neocortex. Later stages (E15-E17) would be suitable if the experimenter wished to target progenitors that produce superficial layer cortical neurons or, in the case of the hippocampus, progenitors that are primarily gliogenic. Rat embryos may also be used, which are larger in size and delayed in development compared with mouse embryos. In these cases, paddle electrodes of appropriate diameter to cover most of the telencephalon should be used (1, 3, 5, or 10 mm) depending on the size of the embryonic brain.

The combination of ex vivo electroporation followed by dissociated cell culture allows a range of factors to be tested, including transcription factors, miRNAs, and epigenetic regulators. Cell-extrinsic factors such as substrates, cytokines, growth factors, or signaling molecules can also be tested, and pharmacological agents can be used to perturb the system. In summary, this protocol offers several advantages over in utero electroporation for examining questions such as the regulation of the hippocampal neuron-glia cell fate switch at embryonic stages.
